# Complex life forms may arise from electrical processes

**DOI:** 10.1186/1742-4682-7-26

**Published:** 2010-06-24

**Authors:** Edward C Elson

**Affiliations:** 1 Department of Electrical and Computer Engineering, University of Maryland, College Park, College Park, Maryland 20742, USA

## Abstract

There is still not an appealing and testable model to explain how single-celled organisms, usually following fusion of male and female gametes, proceed to grow and evolve into multi-cellular, complexly differentiated systems, a particular species following virtually an invariant and unique growth pattern. An intrinsic electrical oscillator, resembling the cardiac pacemaker, may explain the process. Highly auto-correlated, it could live independently of ordinary thermodynamic processes which mandate increasing disorder, and could coordinate growth and differentiation of organ anlage.

## Introduction

### Biology and the second law of thermodynamics

Goldbeter [1] has classified some of the main biological cycles (rhythms) in order of increasing period. Only one, that of the cardiac rhythm, can be regarded as truly governed by a pacemaker in the deterministic sense. The others have large numbers of sub-systems interacting via laws of probability, and although exhibiting "tight" control in some sense, do not possess the high auto-correlation of the cardiac pace-maker, which this paper suggests may be an adaptation of the first pace-maker in evolution, the one facilitating cell specialization (differentiation) as well as proliferation.

The proposal of this paper is that the fundamental cycle supplies the coordination and disciplining of the growth process which the myriads of stochastic biochemical cycles cannot. The fundamental cycle can be thought of deterministic and therefore may explain how development occurs, even though biological organisms remain subject to the second law of thermodynamics.

In what sense does growth and differentiation of a biological organism violate the second law? This is perhaps intuitively apparent, but one should try to make the concept more concrete. It is known that in an isolated box filled with Avogadro's number of gas molecules at equilibrium, the probability that all of the molecules will go in the same direction simultaneously (will "fall up") is very low, even unlikely to happen during the presumed age of the universe. If one places a permeable membrane across the middle of the box, the probability that all molecules will be found in one compartment of the box at a later time is also very low. Likewise, the probability that a lowly protist, say, a bacterium assembles itself from complete, nutrient medium is similarly low. It is known that, over millennia, such a process has occurred, but this article inquires no further about that process, as it pertains to the origin of life, a large and even now incompletely understood process, beyond the scope chosen by the author. Rather this paper addresses the process of replication/differentiation, made possible by the evolution of the eukaryote structure. Nor does this presentation choose to address the problem of how a protist, in a nutrient medium, under the right physical conditions, undergoes fission to form a second, identical protist. This would appear to involve an increase in free energy (the cell plus the universe) and also poses an unsolved problem to efforts to explicate life in purely chemical and physical terms.

In the situation of interest here the desire is to examine the ability of a zygote to commence the process of growth simultaneous with differentiation. Such a cell is far from equilibrium; it is, in fact, in a very dynamic state, exhibited by processes of energy utilization-respiration, anabolic and catabolic processes, with absorption of nutrients and excretion of catabolic products. The zygote is in a dynamic, steady state, but one must focus on what is meant by a steady state in biology as opposed to chemistry. In chemistry molecular species may have the same relative and relatively unchanging concentrations within a defined volume, although far from equilibrium, accepting energy and matter from outside and transferring energy and matter to the outside. In biology and in this study, the defined volume will be called a biological cell which can accept energy and molecular species from a surrounding bath and reject molecular species into the bath similarly keeping the relative concentration of molecular species constant as well as the structural components, organelles, membranes, cytoskeleton, etc. preserved, unchanged.

### The Gamete as Starting Point

One now identifies more concretely the biological cell as a gamete which develops from primordial germ cells that have been set aside during early embryogenesis. One could consider this cell as an example of one kind of the previously mentioned protist whose origins stretch back through geologic time. Such origins are not within the scope of this study. This particular protist, unlike primitive single-celled organisms, is housed within a metazoan structure, is a eukaryote, has no internally originated program of development, and is altered only by external signals. Importantly, it will unite with a homologous gamete to create the instructions for internally directed development. In mammals such germ cells have an extragonadal origin and migrate to reach the somatic gonad where they proliferate by mitosis to form oocytes. Daughter cells of such mitoses are replicas of the parent cells, exhibiting no compositional changes or structural changes initially. At some point an extracellular signal causes such cells to enter meiosis and then be arrested at the prophase of the first meiotic division. This arrest may last years in mammals. During this period, these largely dormant cells accumulate large quantities of mRNA which will later facilitate the oocyte's reentry into a second meiosis (at the time of sperm entry) and control the rate of mitosis during the cleavage divisions after fertilization.

As opposed to the previously mentioned protist, which, through the activation of replicative, transcriptional and translational cascades, new, but compositionally identical progeny are formed, germ cells undergo compositional changes, explained by the extracellular or maternal/paternal signals which, through ligand-binding, are unlocking new translational cascades in the germ cells. The completion of the first meiosis is caused by an extracellular signaling molecule, a gonadotropin in mammals, which sets off a series of biochemical cascades underlying nuclear and cytoplasmic changes moving the oocyte to metaphase of second meiotic division, when oocytes become arrested for a second time and remain in a second "steady state" until fertilization. Once the signaling molecule has activated previously non-functioning pathways the processes can be considered to be stochastic in nature and proceeding generally with a decrease in free energy. In the later stages of oocyte maturation, in mammals there is an absence of new transcription [2] As a result completion of the meiotic cell cycles, reprogramming of the genomes contributed by the egg and sperm, and later activation of the embryonic genome depend entirely on transcripts and proteins made during oocyte growth and on external signal transduction which also programs ovulation. All of these processes start with external signals producing "new" regulatory cascades in which "new" transcription and translation occurs.

Not until the first division of the zygote does any discernible, zygotic transcription occur. McLay and Clarke [3], summarizing earlier reports, state that "in mice transcription by RNA polymerase II is first observed in the late one-cell embryo, initially from the paternal pronucleus, and is followed by the major activation of the embryonic genome at the two-cell stage. Likewise, transcripts of Y-linked genes...are present in one-cell human zygotes, and expression from paternal copies of autosomal genes is observed at the time of activation of the human embryonic genome, the four-cell stage." Knowles et al. have identified approximately 5500 individual genes expressed in the fully grown oocyte library and 4000 in the two cell embryo. About 10% of the genes in the fully grown oocyte library and less than 5% of the two-cell stage library appear to be unique to oocytes and preimplantation stage embryos and suggest that the oocyte to embryo transition is accomplished by interaction of a few stage-specific gene products.

### The Zygote is the Beginning of Autonomous Development

One steps back at this point to make a general observation. It would be trivial to observe that fertilization confers a new set of capabilities upon the oocyte, now identified as the zygote. The sperm has supplied half the genome and the centrosome, an organelle required for the cell division cycle. The sperm also triggers the cortical reaction (block to polyspermy) and the repetitive fertilization-associated calcium transients which have been attributed to the sperm factor phospholipase C-zeta in mammalian species. The capability of replication coordinated with differentiation is, for the first time, contained in the zygote.

With the combined genome the process of transcription begins. The nascent embryo becomes independent of environmental signals and is characterized by autoregulation and self-sufficiency, as the process of dividing and differentiation appears to be programmed "from within" the embryo. Simultaneously there is a decrease in the abundance of receptor and ligand-encoding transcripts [2]. Oocyte polysomes are enriched for transcripts encoding proteins utilized in cellular homeostasis, but zygotic polysomes contain a larger proportion of transcripts used in macromolecular biosynthesis including those functioning in cell cycle regulation. Cui et al. [4] report up-regulation of genes for cytoskeletal, cell adhesion, and cell junctions in the morula as compared to the four cell-stage embryo and suggest that they are involved in the cell compaction essential for the development of the morula,

## Electrical Processes-Voltage-Operated Channels

### Oocytes Evolve in Response to External Signals

The presentation steps back slightly to concentrate more specifically on the changes in calcium channels, calcium ion flow through such channels and changes in membrane voltages around the period of the transition from fully grown oocyte and spermatozoan to fertilized egg. The dynamics of such changes vary vastly with species and one can pick out just a few observations in order to highlight a more general observation. Along with mature somatic cells, such as muscle cells, endocrine cells, and glial cells, fully matured germ cells express voltage dependent ion channels. When such channels arise and, in large part, what function they serve is not known except in a few instances. Cumulus cells and oocytes in mammals maintain different membrane potentials. The cumulus cells transmit stimuli to the oocyte through L-type voltage operated calcium channels on the oocyte plasma membrane, serving a signal-transduction function, setting off translation initiated cascades which ready the oocyte for fertilization. The L-type channels have been shown to underlie meiosis resumption in several species including the mouse. This occurs through complex primary changes in membrane potential which may be driven by phophoinositide metabolism [5]. The exact role played by membrane potential modifications in oocyte maturation is not clear. A T-type low voltage-activated Ca^2 + ^channel is detected in mouse spermatogenic cells by whole cell-patch clamp methods. ZP3, a glycoprotein in the zona pellucida, depolarizes sperm membrane potential from about -60 mV to -25 mV upon contact. This appears to be due to the activation of voltage-insensitive ion channels. This degree of depolarization is insufficient to activate the acrosome reaction, but the major function of the inward currents that do occur is to depolarize sperm membrane potential and thereby open voltage-sensitive T-type Ca^2 + ^channels which are needed for the acrosome reaction [6]. As noted earlier right after fertilization in mammals there is a train of intracellular Ca^2 + ^oscillations accompanied by membrane potential oscillations which continue up to pronucleus formation. In the hamster and bovine systems a clear dependence between membrane potential activity and Ca^2 + ^levels in the cell is found [5]. It is currently believed that the sperm factor phospholipase C activates the cycling of phosphoinositide metabolism underlying the calcium cycles in mammalian systems. Voltage operated Ca^2 + ^channels mediate in some way the operation of the cycles. To the extent that these cycles can be said to have "periods" they can be measured from a minute to tens of minutes, depending on species and various stages of development within a given species [7]. In addition, there are, depending on species, modulations of intensity of calcium waves and changes in resting potential, all of which are thought to be ways in which calcium changes are decoded by specific metabolic processes by mechanisms which are poorly understood.

### The Cardiac Sinoatrial Node Exhibits Automaticity

The analysis will come back later to the role of calcium waves in a variety of cellular and developing embryonic functions, all of which participate in stochastic processes, some of which occur only once in embryonic development and some of which participate in recurring processes, of which the cell cycle is the most prominent. Instead the study refers to repetitive, highly auto-correlated membrane voltage depolarizations identified as action potentials. Calcium flows play a key role but such a process is fundamentally different from the much more slowly evolving calcium flows, in some cases involving membrane depolarization and in other cases not, which have been described. The locus of such a process is the sinoatrial (SA) node, which, in an adult human drives the heart rate, at rest, at about 72 beats per minute. Electrical activity of the SA node can first be discerned in the rat embryonic heart at the three somite stage using voltage-sensitive optical methods [8], followed shortly by the initiation of contraction. In the chick embryo spontaneous membrane depolarizations of SA node cells are observed in the pre-fused cardiac primordia at the six and early seven somite stage. Contractions are observed in the middle period of the nine somite stage [9]. Through extensive observation Kamino observed that the pacemaking area remains at essentially a constant size, comprising perhaps 60 to 150 cells throughout the 7 to 9 somite stages, suggesting that a population of pacemaking cells, instead of a single cell, serves as a rhythm generator in the embryonic chick heart. Initially the action potential-related optical signals are sporadic, then becoming more coordinated with a low frequency. The frequency increases in the chick embryo from the six to the nine somite stage, becoming very regular by the nine somite stage. In aggregations of isolated cardiac cells and in intact embryonic hearts gap junctions are found. Gap junctional conductance is observed mediated by flows of intracellular calcium through the junctions. The initial incoherent and isolated depolarizations of isolated single or clustered SA node cells appear to become collectively coherent through a process of entrainment.

An SA nodal action potential is divided into three phases: The first (arbitrarily designated) phase is the depolarization that triggers the action potential once the membrane potential reaches threshold between -40 and -30 mV. The second phase is the completion of the depolarization phase, followed by the third phase, repolarization to about -60 mV (human values). The changes in membrane potential are brought about by changes in the flow of ions (mostly Ca^2 + ^and K^+^) across the membrane through ion channels that trigger to open and close at different times during the action potential. An open channel is equivalent to increased electrical conductance of specific ions through that channel. Closing of the channel is equivalent to decreased ion conductance. The changing flows through open channels change the membrane potential. The membrane potential can be recorded as a periodic wave (of slightly greater than 1 Hz, resting, in the human, but possessing higher or lower frequencies depending on species. Satoh [10] has described pacemaker activity of the SA nodal cells as a pendulum movement between depolarization and repolarization which continues every moment of the life of the organism from the beginning activity of the pacemaker onward. One can put the process in electrical terms. The pacemaker resembles an electrical oscillator, which, undisturbed in its periodicity, can act as a clock and can be described as highly autocorrelated. The timing of the appearance of voltage-activated channels during the development of the embryo is a function of the timing of gene expression of the components of the channels. It will be argued later that a fundamental pacemaker, from which has evolved the SA node pacemaker, is responsible for the timing of the expression of genes whose products form the structure of the voltage-operated channels of the SA node.

One must make another important distinction between the action potentials of a pacemaker structure and the slower alterations in membrane potential which produce calcium waves both in the embryo and in adult structures. The ions that give rise to the membrane potential lie in a thin (< 1 nm) layer close to the membrane surface, held there by counterions on the other side of the membrane in a configuration maintained at rest by electrogenic pumps producing a potential which can be calculated from the Nernst equation. The movement of 6000 Na^+ ^ions across 1 μ^2 ^of membrane would carry sufficient charge to shift the membrane potential by about 100 mV [11]. There are about 3 × 10^7 ^Na^+ ^ions in 1 μ^3 ^of bulk cytoplasm, so that the movement of charge at the membrane has a negligible effect on the bulk charge in the cytoplasm. One can conclude that if the cytoplasm is, in a gross sense, overall electroneutral (which, of course, is not true at a molecular level), then the interior of the cell could see a gross potential gradient which reflects the status of the membrane potential. Hence, if a depolarization evolves in real time from a micro-locus of the cell membrane and proceeds to spread around the cell, one can envisage a hemisphere of the cytoplasmic side of the membrane as being at a certain time positive while the unpolarized remaining hemisphere on the cytoplasmic side of the membrane is still negative. The cell is then roughly pictured as a "good" capacitor possessing a potential gradient. This is only the case for rapid action-potential-like fast processes. In the case of slower depolarizations which are accompanied by calcium waves (a complex process involving interior organelles such as the endoplasmic reticulum and complex feedback mechanisms) the situation is entirely different. The calcium waves tend to dissipate or cancel out internal potentials, and the concept of the cell as capacitor is not appropriate, or, at least, the cell is a "leaky" capacitor. The generation of an internal potential will be important in the effort to build an electrical model of growth and development.

## An Original or Primal Embryonic Pacemaker

### The Postulate

A key piece of the model is proposed at this point, which is considered testable. **It extrapolates from bits of data **which do not themselves point inexorably to the postulated model. It is proposed that a "primal" pacemaker process starts as early as the zygote with an entrainment similar to that of the earliest formation of the SA node pacemaker, producing action potentials, and with a frequency approximating that of the later-developing SA node. In order to distinguish this "pacemaker process" from the cardiac pacemaker the paper identifies this process as a "primal pacemaker" arising with the organization of the zygote, and made possible only by the presence of both male and female haploid genomes and their associated transcripts and/or proteins. (The biochemistry associated with the primal pacemaker may later be modified in the cardiac anlagen and specialized to become the cardiac pacemaker.) Before these assertions are fitted into the larger model one must explain why such a phenomenon has not been observed, or at least appreciated.

### Measurements and the Ability to Find the Primal Pacemaker

Measurements of membrane potential fall under two general techniques: microelectrode measurements, and optical measurements. Microelectrode measurements have generally been considered more sensitive and quantitative. Optical measurements, an active area of investigation using voltage-sensitive dyes, have included fluorescence, absorption, dichroism, birefringence, fluorescence resonance energy transfer, nonlinear second harmonic generation, and resonance Raman absorption [12]. Most work has been with fluorescence or absorption, but all methods can follow changes in membrane potential with time courses that are rapid compared to the rise time of an action potential. There are problems with sensitivity and quantitation, although second harmonic generation may, in the future, successfully address those problems [13]. A major advantage of optical techniques is the ability to look at voltage changes of whole populations of cells in real time as opposed to microelectrode measurements. It is not certain whether state of the art optical techniques can find our postulated embryonic electrical rhythm, but a review of the literature to date does not find any studies using optical methods on the embryo specifically. This is in spite of the fact that a a few older studies using microelectrode techniques on zygotes and early embryos [14] found spontaneous and repetitive action potentials in fertilized eggs of the tunicate Clavelina, with peak depolarizations of + 10 to + 20 mV and spike durations of 0.6 to 16.8 sec lasting over a period of 10 to 20 minutes. In some cells spontaneous repetitive action potentials occurred at a regular low frequency, firing up to 20 spikes over a 10 to 20 minute period. In other experiments spontaneous action potentials in blastomeres from the two cell stage to the 16 cell stage and up to the early gastrula stage were observed. Nakajo and Okamura [15] studied the blastomeres of the ascidian Halocynthia roretzi and reported that 72 hours after fertilization cleavage-arrested blastomeres showing precoursers of muscle cells exhibited an oscillatory membrane potential at 15 Hz and 48 hours after fertilization a spiking pattern (microelectrode study). Hirano, et al. [16] observed action potentials in the blastomeres of 8 to 32 cell embryos in the same ascidian model. A search of the literature to date suggests that such observations, which go back to the early 1980s have not been followed up with the application of optical techniques. The laboratories specializing in optical monitoring of membrane voltage changes understandably have focused on neural and cardiac processes. In addition there has been no attractive model to direct attention to electrical processes in early embryonic systems. It is very possible that the studies using the approach of Kamino et al. [9] and Sakai [8] who studied the earliest appearance of electrical activity in the chick embryonic pacemaker, using the merocyanine-oxazolone dye NK2367 would be informative.

### Observations Motivating the Search for a Primal Pacemaker

Returning to findings in the literature which suggest a search for an embryonic pacemaker, we look at an interesting cell culture system employing fibroblasts, non-mature cells of mesenchymal origin which can be considered to be related to embryonic cells. Using normal rat kidney (NRK) fibroblasts, De Roos, et al. [17] seeded clonal fibroblasts, grown to density arrest, which then exhibited repetitive intracellular Ca^2+ ^spikes that appeared to be synchronized throughout the entire monolayer. The spikes consisted of a fast rising phase, followed by a slow, declining plateau phase of about one minute, after which levels rapidly declined to resting values, and the spikes recurred about every five minutes in appropriately prepared culture systems. In other systems the frequency ranged from 1 or 2 spikes per 20 minutes to as high as 10 spikes per 20 minutes. It is of interest that the spikes were paralleled by membrane depolarizations, observed by a fluorescent probe as well as by patch clamp measurements. The latter measurements showed that the membrane of a reacting cell depolarizes in an all-or-none, action potential-like manner, from a resting potential of about -50 mV to a peak value up to +20 mV, followed by a fast repolarization to about -10 mV, followed by a plateau characterized by a slower repolarization to about -20 mV. A number of related studies revealed that Ca^2+ ^plays the dominant role, although other ion flows through the membrane also participated in a coordinated fashion.

De Roos et al. [17] proposed that the initiation of the spikes occurs randomly in the monolayer. There is a depolarizing trigger whose nature is not known which causes one focus of the monolayer to depolarize to the threshold value of L-type Ca^2+ ^channels, after which a propagating action potential spreads from the focus. In a separate study by the same authors (De Roos, et al. [18]) it was found that from a starting locus, action potentials travel at a speed of about 6.1 mm/s and depolarize millions of cells. The authors comment that Ca^2+ ^action potentials provide a mechanism for intercellular communication "that is several orders of magnitude faster than the reported Ins (1,4,5) P_3_-dependent intercellular Ca^2+ ^waves in fibroblasts and non-excitable cells (20-50 μ/s, although slower than action potentials in nerve (1-100 m/s) or muscle (0.1-1 m/s).)" "Fibroblasts can also be electrically coupled to other cells, and in this way networks of fibroblasts may play a central role in the synchronous or coordinated behavior of tissues. For example, fibroblasts are electrically coupled to myocytes and can transmit action potentials..."

In a experimental system using vasomotion in lymphatics Imtiaz et al. [19] investigated the activation of endothelin 1 (ET-1)-induced lymphatic pacemaking, which induced transient depolarizations, followed by loose synchronization to form pacemaker potentials, Global synchronization then occurred, mediated by pacemaker-potential transmitted signals, which, when near threshold, triggered action potentials. In this case a chemically mediated process, the application of ET-1, caused enhanced synthesis of IP_3 _which caused Ca^2+ ^store release into the cytoplasm which then led to global synchronous Ca^2+ ^transients associated with action potentials. Kusters et al. [20], using the NRK system of fibroblasts, emphasized that the rapid calcium wave propagation is "boosted" by calcium-induced calcium release (CICR). IP_3_-mediated calcium oscillation and action potential generation couple with each other, producing very robust pacemakers for propagating signals through the cell network.

Dernison et al. [21] showed that NRK fibroblast cultures, stimulated by prostaglandin F_2alpha_, resulted in the induction of periodic action potentials. In this set of experiments it was possible to identify pacemaker cells and establish a relationship between such cells and follower cells. Pacemaker cells, which phenotypically could not be distinguished from follower cells, could be "created" by local application of PGF_2alpha _which resulted ultimately in the transmission of signals to follower cells which themselves exhibited coordinated propagating waves of intracellular Ca^2+ ^and simultaneous propagating action potentials. There is evidence from this study that gap junctions play a critical role, providing not just an intercellular diffusion pathway for IP_3 _but an electrical coupling as well. Electrical current through the gap junctions, driven by the membrane potential difference between the cells is the vehicle for the rapid transmission of the action potential from one cell to the next. (There is a likelihood that the mechanisms which produce transient, pulsed gradient potentials across a cell and from one cell to another may also be employed to set up static gradient potentials extending over sizes on the order of the size of the embryo, and which may drive the electrolyte currents reported in Nuccitelli [22], Levin [23] and others.)

A complex set of membrane voltage changes occurs during gamete maturation and fertilization in eukaryotic, metazoan systems. Following a long-lasting quiescent phase during which gametes undergo a period of growth and maturation, there ensues a series of increasingly rapid biochemical processes associated with ion current flows through plasma membrane channels, changing especially intracellular calcium levels. The ion channels can open in response to ligand binding, stretch-activation, or in response to voltage change. This study is primarily interested in voltage-gated channels [4]. The appearance and functioning of the voltage-gated channels is a first indication that gametes are undergoing activation steps during maturation and fertilization. Shortly after sperm-egg fusion a fertilization current and a large hyperpolarization or depolarization of membrane potential occurs related to the initiation of embryo development. The dynamics of membrane potential changes in relation to oocyte maturation are not clear but a connection to specific events of meiotic maturation and to preparation of the oocyte for fertilization is observed. The fertilizing ability of the sperm depends on a final maturation process known as capacitation, also connected to changes in membrane potential, just before the acrosome reaction upon contact with the egg, which also involves voltage-activated channels and changes in the resting membrane potential. In mammals a series of electrical oscillations of the membrane potential, coordinated with Ca^2+ ^oscillations continue up to pronuclear formation and then decrease in frequency and amplitude [14].

This information is presented in order to illustrate the importance of electrical events surrounding the processes of zygote formation. It was proposed previously [24] that the fusion of sperm and egg brings together a protein or proteins from the separate gametes to start a cycle of regular action potential-like spikes which will orchestrate coordinated growth and differentiation of the embryo. As indicated earlier the fusion may bring together a "lock and key" combination of proteins which can activate, through DNA binding, the necessary expressed genes for the synthesis of voltage-activated ion channels, which can also participate in the cooperation or entrainment necessary to establish the regular rhythm of a pacemaker.

Other observations which suggest some sort of primary oscillator following fertilization focus on post-fertilization calcium oscillations in different species. In Xenopus, cell cycle calcium signals that correlate with mitosis in control embryos persist after the cell cycle is blocked with colchicine to prevent mitosis [25]. This finding implies **that the calcium signaling system is a primary oscillator rather **than an element of a complex feedback system. A similar theme is taken up by Keating et al. [26] who report regular calcium oscillations in normal and cleavage-blocked embryos. In this case a sinusoidal oscillation in intracellular Ca^2+ ^occurs that has the same frequency as cleavage, with cleavage occurring when intracellular calcium is lowest. Although this is a different situation from the much shorter period oscillations of a proposed primal oscillator, the fact that the calcium oscillations persist when nuclear division is not occurring suggests that such oscillations operate independently of any downstream events that they might control. Similarly Swanson et al. [27] report that "transient elevations in the concentration of free cytosolic calcium ion...promote cell phase transitions in early embryonic division and persist even if these transitions are blocked. These observations suggest that a Ca^2+ ^oscillator is an essential timing element of the early embryonic "master clock". One notes as well an observation of Whitaker [25] that calcium transients are observed very early during the cell divisions of the blastomeres and the blastodisc and persist until the sphere stage. Using aequorin luminescence one sees correlated spikes in adjacent cells having the appearance of intercellular calcium waves that propagate through gap junctions. These observations at least raise the question of the existence of a related and perhaps more fundamental electrical oscillator. Beyond even that observation this paper suggests that with fertilization nature has brought together the pieces making possible **a transition from stochastic to deterministic behavior **in a biologic system through the use of a master clock to order growth and differentiation temporally.

Such a phenomenon is envisioned as a locus of one or a few cells which transmits a repeated electrical signal to all cells of the early embryo, and which results in a pulsed gradient of potential across each cell with each signal, with a repetition rate which we provisionally suppose is on the order of magnitude of one or a fraction of a Hertz.

### Direct Observations on the Zygote and Early Embryo

Is there any evidence that a momentary or pulsed and repeating gradient of electrical potential occurs across non-excitable cells? The thinking is directed, in particular, to the early cells of the embryo from the zygote on through the processes of blastula and gastrula formation, embryonic germ layer formation and organ system formation. It is known that there are more or less steady potential gradients at the cellular level which combine into supra-cellular gradients and which are motivating forces in the migration of cells and cell layers in embryonic growth processes as well as forces producing ion-based currents coursing through and around the surface of embryos. Little is known about the origin of the gradients or the function of the embryonic and extra-embryonic currents. The pulsed process proposed here is different from the establishment of these quasi-steady currents, which are orders of magnitude larger than any proposed DNA currents. Such steady currents are associated with gradient potentials on the order of the size of embryo [22].

No studies have shown that the continuous cycle of pulses as described above occurs. Many investigations using micro-electrode techniques and optical visualization of voltage sensitive dyes have been performed on developing oocytes, fertilized eggs, and then mature, adult forms of many metazoan systems, but virtually no studies have been done on systems evolving beyond the first or second mitoses following fertilization. The studies which have been done show a discernible pattern of membrane voltage changes during gametogenesis, and fertilization which can be related to developmental changes but not with spikes or action potentials. Measurements from microelectrode studies would detect only polarization changes of the entire plasma membrane, but not focal or domain polarization of one small part of the plasma membrane, a process associated with calcium puffs. The puff phenomenon is described below in detail and can be a source of a cellular gradient potential as argued below.

This information is presented in order to illustrate the importance of electrical events surrounding the processes of zygote formation. It was proposed previously [24] that the fusion of sperm and egg brings together a protein or proteins from the separate gametes to start a cycle of regular action potential-like spikes which will orchestrate coordinated growth and differentiation of the embryo.

### The Possible Function of a Primal Pacemaker

What might such a process be used for? In the following paragraphs the presentation argues that an embryonic pacemaker might act as a beacon, transmitting a regular pulse to the cells of the developing embryo, causing action potentials in every cell more or less simultaneously, thereby governing gene expression through the generation of certain seminal proteins which stand at the head end of nascent biochemical cascades or cycles. Some of those seminal proteins may, in fact, be proto-oncogenes, a concept pursued later in this presentation.

One now examines the effect of such a putative beacon on each of the myriad of embryonic cells as the electrical impulse it generates spreads through embryonic tissue. The argument is that the impulse produces a spatial gradient of potential across each cell in its path, a path which may be more like an outwardly radiating wave from the pacemaker. This argument paves the way for the role of DNA current conduction which is subsequently discussed. It is considered whether such an "action potential" might actually occur, and then one looks at how the coupling or transduction of potential energy into current flow in DNA might actually occur.

There is no direct evidence of "spike" potential changes with a spatial gradient across an individual cell. This paper marshaled some circumstantial evidence by reporting propagating action potentials in certain experimental systems. The survey of the literature does not find data to support this model, but no experiment appears to have been done to look for such a process. Such an experiment would be fairly challenging and could only be motivated by an attractive physical model. As reported earlier Dernison et al. [21] described the transmission of signals from pacemaker to follower cells in the NRK fibroblast culture system, and suggested that the signal propagates from one cell to the next as a wave of depolarization advances along the plasma membrane and sets up an electrical current or a depolarization right through the gap junctions. This suggests that, as the depolarization spreads over the surface of a cell in real time, an electrical field across the cell directed from the momentarily positively charged inner side of the plasma membrane to the still negatively charged inner side of the plasma membrane exists. The implication of such a process was explored in Elson [24]. Using experimental data applied to a biological cell of "average" dimensions, and assuming a potential drop across the nuclear envelope (in electrical continuity with the plasma membrane through the endoplasmic reticulum) of 15 mV, it was estimated crudely that a field of about 50 V/cm was possible. This is in the same range as that described in the *in vitro *experiment of Cohen et al., [28] in which current/voltage curves through 26 mer DNA suspended between electrodes have been recorded.

There is another phenomenon which could account for a potential drop across the nucleus, and that is the existence of rapidly appearing and disappearing calcium microdomains, that is, sub-membranous discrete intra-cytoplasmic high-calcium foci associated with activation of voltage and ligand-gated plasma membrane ion channels [29]. Such focal domains, also known as puffs, sparks, blips, or quarks, have been investigated in relation to a number of activities as a second messenger. They are of interest in the context of this presentation because they offer the possibility of producing the desired potential drop across the nucleus by a mechanism different from that described in the previous paragraph. In this case a momentary focal concentration of intracytoplasmic calcium can produce a capacitance with the resting membrane voltage on the side of the cell opposite the micro-domain without postulating a wave of depolarization spreading from one focus around the surface of the cell. As a signal propagates across a tissue its leading edge can activate micro-domain formation through a subset of adjacent voltage operated channels. Assuming a repeating or cycling process the description would be consistent with the observations of Silver [30] on blastomeres in the sand dollar, namely, that microdomains appear in a cyclic, programmed fashion, closely coordinated with events of the cell cycle.

### Effect of Pulses on the Genome

The possibility of an electric field transient is now connected to the ability of DNA strands to act as a "molecular wire" having the ability to conduct the passage of charge. This idea is an extrapolation from a corpus of *in-vitro *experimental data [31]. There is no generally accepted evidence that such a process occurs *in-vivo *or that it might serve a biological function, but there is speculation that such a process could serve a protective function *in-vivo*. One thought has been that long distance hole transport through DNA might protect genes from oxidative mutations, in that several genes possess GC-rich sequences outside of the coding area. Such sequences can be sinks for positive charge [32,31]. At least two published papers have suggested that such currents, if they existed, could produce internal forces on DNA causing individual strands of a double-helix to separate from one another [33,24]. The latter paper, using elementary electromagnetic theory showing that parallel currents in adjacent strands set up repulsive forces, has proposed that such a process could influence gene expression and replication by producing origin sites for replication and transcription, and is consistent with the observations of Heng [34], Bode et al. [35], and Bode et al. [36] on the location of origin and transcription start-sites at SIDD-determined loci (see below). If such currents exist there is the remaining problem of whether they could produce the forces necessary to actually cause localized strand separation [24]. Here it is postulated that DNA currents can be generated in an individual cell through localized or cell-wide propagation of membrane depolarization, resulting in a momentary gradient of potential across the cell, and involving the connected DNA-nuclear envelope-nucleoplasmic reticulum-endoplasmic reticulum-plasma membrane system, a system for which there is anatomic and functional evidence [37]. It must also be reported that there is anatomical evidence for a connection of chromatin to the nuclear envelope. On the inner (nuclear) surface of the NE is the nuclear lamina, a network of filaments physically connected to the telomeres and centromeres of DNA [38]. The same filaments extend into the fibrillar network in the interior of the nucleus as part of the nuclear scaffold. Loops of chromatin in between telomeres can be envisioned as being in contact with potential differences arising from the depolarization wave.

There is a question of whether there would be enough energy in such a process to produce the currents which mechanically could separate the strands of the double-helix. The issue of sufficient energy is addressed in Elson [24] in which a case is made that sufficient energy is available consistent with the SIDD model. The mere calculation of the strength of the bonding between the strands of double helical canonical, Watson-Crick DNA in solution does not mean much in and of itself without a consideration of the milieu of genomic DNA *in vivo*.

To set the stage for a consideration of force and compliance *in vivo *it is noted that for replication or gene expression to commence something must separate the DNA strands and hold them apart for some finite period of time to allow recognition proteins involved with either process to begin or continue the cascade of processes constituting the mechanics of replication or gene expression. Furthermore, the locations where separation occurs in the genome cannot be random, but must be specialized to biologically relevant loci. One observes that replication and transcription foci appear to originate in or adjacent to scaffold/matrix attachment regions (S/MARs), and involve the same or overlapping regions of DNA [34-36]. A replicator is defined as the entire set of cis-acting sequences that are minimally necessary to cause the initiation of replication including the exact point of origin of DNA bubble formation. Within the replicator an intricate succession of proteins is or becomes attached, starting with the origin recognition complex (ORC). Within this succession is a single-strand binding protein (SSB), Replication Protein A (RPA), referred to below, which will detect a length of suddenly available single-stranded DNA and which acts in a permissive fashion to allow the process of replication to unfold. The same concept may also explain the way in which promoters and enhancers could act, namely, through the separation of a segment of H-bonded base-pairs allowing the interpolation of a single-strand binding protein (SSB) which facilitates down-stream gene expression, an example of which is the far-upstream binding protein also described below. An argument will be developed later that the unique specificities of the gene regulator proteins allow individual genes to be turned on or off specifically, and especially with regard to cell differentiation, confer the patterns of gene expression that give each cell type its distinctive set of proteins and phenotype.

It was observed that the base sequence in the replicator possesses common elements, especially AT-rich sequences, making H-bonding across base-pairs aggregately weaker, and containing transcription factor binding sites and sequences which, under torsional stress, undergo strand separation, facilitating the initiation of transcription and/or replication. In these regions of non-coding DNA segments of the duplex are de-stabilized in the sense that under conditions of mild stress, in the presence of superhelical strain, strand separation occur much more readily than in the remaining regions of DNA. These segments are called unpairing elements (UEs) [35,36]. Benham [39-41] developed an algorithm called Stress-Induced-Duplex-Destabilization (SIDD), which can predict from a number of parameters, including superhelical strain, the locations of UEs as a function of base-pair sequence. The UEs lie within larger regions that cooperate, within the meaning of SIDD, called base-unpairing regions (BURs). The BURs coincide with S/Mars to a considerable extent. The amount of energy required to separate the strands in these regions **could be vanishingly small, **making a current-induced separation a physical possibility.

Adjacent to or within S/MARs, and related to UEs, are segment of DNA which act as enhancers, promoters, insulators, and ARS-like sequences in eukaryotic cells, and replicators. The coincidence of these elements with UEs is such that these elements can become single-stranded and interact with SSBs. The contemporary understanding of DNA kinetics is that there exists a replicon, the segment of DNA ultimately replicated from a single bidirectional origin and the length of DNA between adjacent S/MARs. Adjacent replicons are themselves part of a functional unit called a replication site, defined as an entity containing 20 to l00 loops. The entire genome is organized into replication sites. Each replication site exhibits activation of all replicons within itself simultaneously in a schedule of activation of such sites which does not vary from one generation of cells to another. The simultaneous activation is very suggestive of a control mechanism of an electrical nature rather than the diffusion of an activating molecule.

Additional evidence is now presented that SSBs play a key role in control of transcription and replication. With regard to the replication associated with the cell cycle the phased appearance and disappearance of replication-associated proteins around the replicator region was previously presented [24]. Around the time of the formation of the replication origin (bubble) Cdc45, replication protein A (RPA), an SSB, and DNA polymerase alpha associate with chromatin. Cdc45 binds just before, RPA "concurrent" with, and DNA polymerase alpha just after helicase-mediated origin unwinding. RPA is permissive and critical. In the model advocated here it becomes functional with the passage of a current pulse. In the model, every round of cell division is influenced by a clock-like response to a pacemaker and therefore subject to coordination. The frequency of response, obviously different for different cell types, would be a function of the state of the genome of a given cell, a point which will be addressed later.

An example of the regulation of gene expression is that of one of the promoter systems regulating the expression of c-myc, which, among other functions, has an integral role in differentiation. The far-upstream binding protein FBP, in a family of SSBs having transcriptional-activation factors, binds to only one strand at the far-upstream element FUSE in a region 5' of the oncogene c-myc, and mediates activation domains when fused to the GAL4-DNA binding domain [42]. Additional examples of transcriptional activators of "master-regulator" proteins employing an SSB mechanism have been described in Elson [24].

How does the transmission of a periodic signal produce coordinated growth and differentiation in a metazoan structure? One can cast the problem abstractly and then describe a few well studied systems which would be candidates for the postulated process. Abstractly, the expression of a protein is affected by proteins which bind to its DNA code-script itself or to promoter and/or enhancer regions of the expressed proteins at other regions of DNA upstream or downstream of the DNA code-script in cis or trans configurations. Replicator, enhancer or promoter regions can be protected or repressed by bound proteins or they can be relatively exposed but capable of expression only by a particular promoter protein. For the sake of simplicity, one can say that, early in the life of the embryo a signal or pulse opens up a given promoter origin, say, of a master-regulator protein, resulting in expression of that protein, The expressed protein can act as a de-repressor of a large number of proteins to activate a coordinated process, such as the cell-cycle or the anlagen proteins of a limb-bud. It may be that among the newly expressed proteins there is an SSB which has been expressed or "awakened" and which will be able to respond to a later signal and act as a master-protein activator which can stand at the head-end of another process, perhaps the anlagen proteins which will subserve the construction of later, more highly differentiated limb-bud structures. What the process would bring is temporal order. Contra-lateral limb-bud structures can develop at the same time. Separate chambers of the primitive heart can develop contemporaneously and those fusion proteins which cause fusion of bilateral chambers into one central tube arise simultaneously. Other organ systems can develop in concert with each other. There is an interaction between pulse and genome, which, in turn, modifies the genomic response to subsequent pulses.

One observes that the postulated pulse contains no information. It spreads through embryonic tissue and produces single-stranded DNA wherever DNA "compliance" exists. This would be at origin sites or promoter sites (in accordance with SIDD, chromatin structure and the presence or absence of repressors at such sites). Following a round of replication and "new" gene expression, the daughter cell(s) have acquired a new dynamic genomic state in which new proteins may execute a program producing a different phenotype, that is, executing a program of differentiation. The original differentiation program occurs with the division of the zygote into two cells with a different complex of DNA binding proteins.

It is still unestablished just when, after the first cell division in mammals, that cells become non-equivalent and exhibit the first signs of embryonic polarity. Some evidence has been presented of differentiation before blastocyst formation but the issue continues to be debated [43]. An earlier paper alluded to a model which suggests differentiation after the first cell division, connected with the binding of gamete-originated zygotic proteins, maternally and paternally derived, to different chromatid-loci which assort into the daughter cells, [24] but there is no phenotypic or biochemical evidence for such early differentiation. In fact the cells of up to eight cell embryos can be teased apart and recombined with cells from another embryo to produce a healthy mouse.

One can next describe specific processes as candidates for governance by the proposed abstract model, just described, through the following identified "master-regulator "proteins (which stand at the head of and set off the sub-routines, so to speak, of the congeries of integrated and interacting biochemical modules which contribute to the growth and differentiation process):

Among them may be cyclin-D which may be electrically activated to forward a cell, destined to form a given tissue, through the latter half of G1 into S phase. Such a regulator is the architect of proliferation. It may act at the Restriction (R) point of the cell to propel the cell into the cell cycle. In G_0 _and early G_1 _the activity of all of the cyclin-dependent kinases (CDKs) is suppressed by the action of high CDK-inhibitors (CKI) and low cyclin levels. Upon the appropriate external signal (postulated to be electrical) D-type cyclins begin to accumulate, overcoming the anti-proliferative activity of the CKI, thus activating the biochemical "sub-routine" of the cell cycle. Early into the sub-routine, set off by increasing Cyclin D, is the phosphorylation of the retinoblastoma protein, pRb, which leads to the release of the E2F transcription factors which cause the transcription of many genes that encode proteins for S-phase evolution. Among those proteins are the set that cluster around DNA replication origins and form "factories" in which the DNA is processed for replication. Part of such a factory is the RPA SSB, ready to respond to the signal to permit the commencement of DNA replication.

Of course there are many other environmental stimuli, operating mostly through ligand binding, for example, diffusible growth factors, extracellular matrix components, and cell-to-cell adhesion/interaction molecules, but these factors are not fundamental directors of development (at least not in embryological development). As the engine of the cell cycle drives forward it may next be subject to a coordination at the signaling-RPA binding point.

If one looks at c-myc as a possible candidate for a master-regulator, there are a bewildering number of binding factors for this gene, which operate on different parts of a complex promoter region [44]. In addition there are numerous feedback loops which activate or repress c-myc transcription, suggesting that it can serve both as master regulator and in homeostasis. c-myc as master regulator is essentially not expressed in quiescent cells. Its expression rapidly rises during re-entry into the cell cycle (G_0_/G_1 _transition), demonstrated *in vitro *by response to mitogens, and possibly by electrical activation according to the proposed model. In the absence of such stimuli or in the presence of differentiation stimuli or other antiproliferative signals c-myc is downregulated. It ceases expression in terminally differentiated cells. Interestingly the c-myc promoter is a target of the Wnt signaling pathway. This pathway is known to be important for embryonic development and proliferation and in promoting stem cell proliferation.

It is the formation of focal segments of single-stranded DNA, in the proposed model created by current pulses, in the c-myc promoter and at replication origin sites, which may qualify c-myc as having master-regulator properties in addition to processing many signaling molecules subserving other functions.

An earlier report [45] identified a protein from HeLa cell nuclei which recognizes specific sequences in a replication origin near the hamster *dhfr *gene. It is an SSB which binds at origins of replication and in gene flanking regions in a range of eukaryotes from yeasts to humans. It binds in a region 1.6 kb upstream of the P1 transcription start site of the human c-myc gene and near the center of a reported zone of initiation of DNA replication, giving it a possible role as cis-activator of transcription as well as replication.

An SSB has been described as interacting with the promoter region of the beta-casein gene promoter and may act as a repressor [45]. It has also been reported that a mitochondrial SSB is required for mitochrondrial DNA replication and development in *D. melanogaster *[46]. An SSB has also been found to interact with a single stranded DNA sequence located in the S1 nuclease-sensitive site of the EGF receptor 5' flanking region [47]. SSBs have also been shown to regulate the presence of LIM domain and LIM domain-binding proteins which are important in developmental programs [48].

### Support for the Role of Current Flows

Lastly, one can observe that there is indirect support for the idea that embryological development is driven by coordinated processes of charge transfer or flows of currents. Reports from the bioelectromagnetics literature show that exposure of sea urchin embryos to 60 Hz magnetic fields at 0.1 mT rms cause significant developmental delays [49]. Complex changes in the mitotic cycle of sea urchin embryos as a function of field strength have also been reported in response to magnetic fields varied from 2.5 to 6.5 mT and frequencies from DC to 600 kHz [50]. Such field strengths are considerably stronger than those found in our technological environment, but such pronounced effects are not found in adult organisms. It is very possible that the time-varying magnetic fields are interacting with the postulated embryological currents and that the lack of effect in mature organisms is due to the lack of such currents beyond a period of embryological development.

## Overall Summary

In two previous papers [24,51] the author has advocated for a model of organism development in which molecular processes interact with and are responsive to electrical processes, some of which are well characterized and some of which are speculative. To drive beyond strictly data-driven models is usually a recipe for rejection for publication based on the general experience that such models are often unproductive or unphysical. On the other hand, the accretion of more and more data has not generated an overall model to explain the deterministic aspect of organism development, to explain, say, the precise end number of cells of different types, in exactly the same relationship to each other, structurally and functionally found in an organism like C. elegans, lowly compared to nature's creatures, but possessed of confounding complexity compared to any inanimate structure. It seems unlikely that the feedback mechanisms of computational biology, capable of description in the language of chemical kinetics, can entirely explain such a process, although no rational argument can exclude such a possibility. The argument of this paper cannot be "proven" by a particular experiment. It has to appeal to a sense of dissatisfaction with the idea that unvarying programs of the development of a species in space and time could be accomplished by inherently probabilistic processes. Instead, the proposition here is that such processes, constituting a major part of development, are undergirded and guided by a smaller number of deterministic processes, more or less fixed at interval points. If no model that is strictly data-driven can suggest a way forward, one can propose a non-strictly-data-driven model, as long as it is not unreasonable or unphysical and, importantly, is testable. This paper offers such a model and erects a set of specifics, some of which separately could be found invalid while not invalidating its more general approach, that is to say, the model is malleable and heuristic, and, with recent technologies, testable.

The idea is that the apparently stochastic nature of organism development cannot by itself alone explain the repeated and unvarying construction of a complex organism of a given species. One observes that after repeated replications of one cell, germ layer formation is followed by organ system formation, but it should be the case that as each organ system evolves its own complex structures there would be small random variations in succeeding generations of regulatory molecules which would themselves produce variations of later regulatory modules, especially that of the cell cycle. Among the, say, 360,000 mRNA molecules in the cytoplasm of one cell there are particular mRNAs that may have only 10-20 copies per cell. These are often critical regulatory molecules, master-regulatory proteins. The lower the number of such molecules, the further a cell cycle is from a deterministic model. It is likely that the presence of large numbers of interacting cyles will have a multiplicative effect on the dis-coordination of populations of cycling cells. With cell cycle and other modules considered, random variations in key regulatory molecules should produce amplified variations in descendant regulatory molecules, resulting in deleterious variations in relative size, relative development, and interaction of organ systems with each other. The transition from stochastic to deterministic behavior in animate and inanimate systems is an active subject of investigation [52].

The model presented here suggests that a periodic signal is produced by a biological structure, which is highly auto-correlated. The structure protects its capability to remain unchanged in time through entrainment of a mass of localized cells, namely a pacemaker structure, which can "defy" the second law, and transmit its signal to a common codescript of all surrounding cells. The process resembles digital logic, amounting to a succession of activated genes which are connected in such a way that they can turn each other on or off, in steps regulated by a clock. The result is the ordered control of replication and gene expression. The model in its entirety is a specific evocation of a set of principles originally proposed by E. Schrodinger in the 1940s [53].

## Competing interests

The author declares that he has no competing interests.

## References

[B1] Goldbeter A Biochemical Oscillations and Cellular Rhythms 1996 Cambridge University Press

[B2] Knowles BB Evsikov AV de Vries WN Peaston AE Solter D Molecular control of the oocyte to embryo transition Phil Trans R Soc Lond B 2003 358 1381 1388 10.1098/rstb.2003.1330 PMC1693239 14511485

[B3] McLay DW Clarke HJ Remodelling the paternal chromatin at fertilization in mammals Reproduction 2003 125 625 633 10.1530/rep.0.1250625 12713425 PMC5123868

[B4] Cui XS Li XY Shen XH Bae YJ Kang JJ Kim NH Transcription profile in mouse four-cell, morula, and blastocyst: Genes implicated in compaction and blastocoels formation Mol Reprod Dev 2007 74 133 143 10.1002/mrd.20483 16998848

[B5] Tosti E Boni R Electrical events during gamete maturation and fertilization in animals and humans Human Reproduction Update 2004 10 43 65 10.1093/humupd/dmh006 15005464

[B6] Florman HM Arnoult C Kazam IG Li C O'Toole MB A perspective on the control of mammalian fertilization by egg-activated ion channels in sperm: a tale of two channels Biology of Reproduction 1998 59 12 16 10.1095/biolreprod59.1.12 9674987

[B7] Swann K Yu Y The dynamics of calcium oscillations that activate mammalian eggs Int J Dev Bio 2008 52 585 594 10.1387/ijdb.072530ks 18649272

[B8] Sakai T Kamino K Optical mapping approaches to cardiac electrophysiological functions Jpn J Physiol 2001 51 1 18 10.2170/jjphysiol.51.1 11295638

[B9] Kamino K Optical approaches to ontogeny of electrical activity and related functional organization during early heart development Physiol Rev 1991 71 53 91 1986392 10.1152/physrev.1991.71.1.53

[B10] Satoh H Sino-Atrial nodal cells of mammalian hearts: ionic currents and gene expression of pacemaker ionic channels J Smooth Muscle Res 2003 39 175 193 10.1540/jsmr.39.175 14695028

[B11] Alberts B Ed Molecular Biology of the Cell 1994 3 Garland Publishing, Inc

[B12] Zochowski M Wachowiak M Falk CX Cohen LB Lam Y-W Antic S Zecevic D Imaging membrane potential with voltage-sensitive dyes Biol Bull 2000 198 1 21 10.2307/1542798 10707808

[B13] Dombeck DA Blanchard-Desce M Webb WW Optical recording of action potentials with second-harmonic generation microscopy J Neurosci 2004 24 999 1003 10.1523/JNEUROSCI.4840-03.2004 14749445 PMC6729824

[B14] Thompson S Knier J Spontaneous action potentials and resting potential shifts in fertilized eggs of the tunicate Clavelina Dev Biol 1983 99 121 31 10.1016/0012-1606(83)90259-2 6684601

[B15] Nakajo K Okamura Y Development of transient outward currents coupled with Ca2+ induced Ca2+ release mediates oscillatory membrane potential in ascidian muscle cell J Neurophysiol 2004 92 1056 1066 10.1152/jn.00043.2004 15056691

[B16] Hirano T Takahashi K Yamashita N Determination of excitability types in blastomeres of the cleavage-arrested but differentiated embryos of an ascidian J Physiol 1984 347 301 325 6323697 10.1113/jphysiol.1984.sp015067 PMC1199448

[B17] De Roos ADG Willems PHG Peters PHJ van Zoelen EJJ Theuvenet APR Synchronized calcium spiking resulting from spontaneous calcium action potentials in monolayers of NRK fibroblasts Cell Calcium 1997 22 195 207 10.1016/S0143-4160(97)90013-0 9330790

[B18] De Roos ADG Willems PHG van Zoelen E Theuvenet APR Synchronized Ca^2+ ^signaling by intercellular propagation of Ca^2+ ^action potentials in NRK fibroblasts Am J Physiol 2007 273 C1900 C1907 10.1152/ajpcell.1997.273.6.C1900 9435495

[B19] Imtiaz MS Zhao J Hosaka K von der Weid P-Y Crowe M van Helden DF Pacemaking through Ca2+ stores interacting as coupled oscillators via membrane depolarization Biophys J 2007 92 3843 3861 10.1529/biophysj.106.095687 17351003 PMC1869001

[B20] Kusters JMAM van Meerwijk WPM Ypey DL Theuvenet APR Gielen CCAM Fast calcium wave propagation mediated by electrically conducted excitation and boosted by CICR Am J Physiol Cell Physiol 2008 294 C917 C930 10.1152/ajpcell.00181.2007 18199705

[B21] Dernison MM Kusters JMAM Peters PHJ van Meerwijk WPM Ypey DL Gielen CCAM van Zoelen EJJ Theuvenet APR Local induction of pacemaking activity in a monolayer of electrically coupled quiescent NRK fibroblasts Cell Calcium 2008 44 429 40 10.1016/j.ceca.2008.02.005 18359515

[B22] Nuccitelli R Endogenous electric fields in embryos during development, regeneration and wound healing Radiat Prot Dosimetry 2003 106 375 383 14690282 10.1093/oxfordjournals.rpd.a006375

[B23] Levin M Large-scale biophysics: ion flows and regeneration Trends Cell Biol 2007 17 261 270 10.1016/j.tcb.2007.04.007 17498955

[B24] Elson E Developmental control in animals and a biological role for DNA charge transfer Prog Biophys Mol Biol 2007 95 1 15 10.1016/j.pbiomolbio.2006.07.001 17011027

[B25] Whitaker M Calcium at fertilization and in early development Physiol Rev 2006 86 25 88 10.1152/physrev.00023.2005 16371595 PMC3299562

[B26] Keating TJ Cork RJ Robinson KR Intracellular free calcium oscillations in normal and cleavage-blocked embryos and artificially activated eggs of Xenopus laevis J Cell Sci 1994 107 2229 37 7983182 10.1242/jcs.107.8.2229

[B27] Swanson CA Arkin AP Ross J An endogenous calcium oscillator may control early embryonic division Proc Natl Acad Sci USA 1997 94 1194 1199 10.1073/pnas.94.4.1194 9037029 PMC19767

[B28] Cohen H Nogues C Naaman R Porath D Direct measurement of electrical transport through DNA molecules Proc Natl Acad Sci USA 2005 102 11589 11593 10.1073/pnas.0505272102 16087871 PMC1188002

[B29] Demuro A Parker I Imaging single channel calcium microdomains Cell Calcium 2006 40 413 422 10.1016/j.ceca.2006.08.006 17067668 PMC1694561

[B30] Silver RB Calcium, BOBs, QEDs, microdomains and a cellular decision: control of mitotic cell division in sand dollar blastomeres Cell Calcium 1996 20 161 179 10.1016/S0143-4160(96)90105-0 8889207

[B31] Merino EJ Boal AK Barton JK Biological contexts for DNA charge transport chemistry Curr Opin Chem Biol 2008 12 229 237 10.1016/j.cbpa.2008.01.046 18314014 PMC3227530

[B32] Giese B Long-distance electron transfer through DNA Annu Rev Biochem 2002 71 51 70 10.1146/annurev.biochem.71.083101.134037 12045090

[B33] Blank M Goodman R A mechanism for stimulation of biosynthesis by electromagnetic fields: charge transfer in DNA and base pair separation J Cell Physiol 2007 214 20 26 10.1002/jcp.21198 17620313

[B34] Heng HH Goetze S Ye CJ Liu G Stevens JB Bremer SW Wykes SM Bode J Krawetz SA Chromatin loops are selectively anchored using scaffold/matrix-attachment regions J Cell Sci 2004 117 999 1008 10.1242/jcs.00976 14996931

[B35] Bode J Goetze S Heng H Krawetz SA Benham C From DNA structure to gene expression: mediators of nuclear compartmentalization and dynamics Chromosome Res 2003 11 435 45 10.1023/A:1024918525818 12971720

[B36] Bode J Winkelmann S Gotze S Spiker S Tsutsui K Chengpeng B Prashanth AK Benham C Correlations between scaffold/matrix attachment region (S/Mar) binding activity and DNA duplex destabilization energy J Mol Biol 2005 358 597 613 10.1016/j.jmb.2005.11.073 16516920

[B37] Jackson D The principles of nuclear structure Chromosome Res 2003 11 387 401 10.1023/A:1024954123092 12971716

[B38] Mattout-Drubezki A Gruenbaum Y Dynamic interactions of nuclear lamina proteins with chromatin and transcriptional machinery Cell Mol Life Sci 2003 60 2053 63 10.1007/s00018-003-3038-3 14618255 PMC11138856

[B39] Benham CJ Sites of predicted stress-induced DNA duplex destabilization occur preferentially at regulatory loci Proc Natl Acad Sci USA 1992 90 2999 3003 10.1073/pnas.90.7.2999 PMC46224 8385354

[B40] Benham CJ Duplex destabilization in superhelical DNA is predicted to occur at specific transcriptional regulatory regions J Mol Biol 1996 255 425 434 10.1006/jmbi.1996.0035 8568887

[B41] Benham C Kohwi-Shigematsu K Bode J Stress-induced duplex DNA destabilization in scaffold/matrix attachment regions J Mol Biol 1997 274 181 196 10.1006/jmbi.1997.1385 9398526

[B42] Davis-Smyth T Duncan RC Zhen T Michelotti G Levens D The far upstream element-binding proteins comprise an ancient family single-strand DNA-binding transactivators J Biol Chem 1996 271 31679 31687 10.1074/jbc.271.49.31679 8940189

[B43] Vogel V Embryologists polarized over early cell fate determination Science 2005 308 782 3 10.1126/science.308.5723.782 15879186

[B44] Wierstra I Alves J The c-myc Promoter: Still MysterY and Challenge Adv Cancer Res 2008 99 113 333 full_text 18037408 10.1016/S0065-230X(07)99004-1

[B45] Bergemann AD Johnson EM The HeLa Pur factor binds single-stranded DNA at a specific element conserved in gene flanking regions and origins of DNA replication Mol Cell Biol 1992 12 1257 1265 1545807 10.1128/mcb.12.3.1257-1265.1992 PMC369558

[B46] Maier D Farr CL Poeck B Alahari A Vogel M Fischer S Kaguni LS Schneuwly S Mitochondrial single-stranded DNA-binding protein is required for mitochondrial DNA replication and development in Drosophila melanogaster Mol Biol Cell 2001 12 821 830 11294889 10.1091/mbc.12.4.821 PMC32269

[B47] Chen LL Clawson ML Bigrami S Carmichael G A sequence-specific single-stranded DNA-binding protein that is responsive to epidermal growth factor recognizes an S1 nuclease-sensitive region in the epidermal growth factor receptor promoter Cell Growth Differ 1993 4 975 983 8117624

[B48] Xu A Meng X Cai Y Liang H Nagarajan L Brandt SJ Single-stranded DNA-binding proteins regulate the abundance of LIM domain and LIM domain-binding proteins Genes Dev 2007 15 942 55 10.1101/gad.1528507 PMC1847712 17437998

[B49] Zimmerman S Zimmerman AM Winters WD Cameron Il Influence of 60-H magnetic fields on sea urchin development Bioelectromagnetics 1990 11 37 45 10.1002/bem.2250110106 2346506

[B50] Levin M Ernst SG Applied AC and DC magnetic fields cause alterations in the mitotic cycle of early sea urchin embryos Bioelectromagnetics 1995 16 231 240 10.1002/bem.2250160405 7488256

[B51] Elson E II. Model building: an electrical theory of control of growth and development in animals, prompted by studies of exogenous magnetic field effects (paper I), and evidence of DNA current conduction, in vitro Electromagn Biol Med 2009 28 283 309 20001704 10.3109/15368370903114297

[B52] Kummer U Krajnc B Pahle J Green AK Dixon CJ Marhi M Transition from stochastic to deterministic behavior in calcium oscillations Biophys J 2005 39 1603 1611 10.1529/biophysj.104.057216 PMC1366664 15994893

[B53] Schroedinger E What is Life? 1967 Cambridge University Press (first published 1944)

